# Disentangling *Peronospora* on *Papaver*: Phylogenetics, Taxonomy, Nomenclature and Host Range of Downy Mildew of Opium Poppy (*Papaver somniferum*) and Related Species

**DOI:** 10.1371/journal.pone.0096838

**Published:** 2014-05-07

**Authors:** Hermann Voglmayr, Miguel Montes-Borrego, Blanca B. Landa

**Affiliations:** 1 Division of Systematic and Evolutionary Botany, Department of Botany and Biodiversity Research, University of Vienna, Wien, Austria; 2 Institute of Forest Entomology, Forest Pathology and Forest Protection, Department of Forest and Soil Sciences, BOKU-University of Natural Resources and Life Sciences, Wien, Austria; 3 Department of Crop Protection, Institute for Sustainable Agriculture (IAS), Spanish National Research Council (CSIC), Córdoba, Spain; Virginia Tech, United States of America

## Abstract

Based on sequence data from ITS rDNA, *cox1* and *cox2*, six *Peronospora* species are recognised as phylogenetically distinct on various *Papaver* species. The host ranges of the four already described species *P. arborescens*, *P. argemones*, *P. cristata* and *P. meconopsidis* are clarified. Based on sequence data and morphology, two new species, *P. apula* and *P. somniferi*, are described from *Papaver apulum* and *P. somniferum*, respectively. The second *Peronospora* species parasitizing *Papaver somniferum*, that was only recently recorded as *Peronospora cristata* from Tasmania, is shown to represent a distinct taxon, *P. meconopsidis*, originally described from *Meconopsis cambrica*. It is shown that *P. meconopsidis* on *Papaver somniferum* is also present and widespread in Europe and Asia, but has been overlooked due to confusion with *P. somniferi* and due to less prominent, localized disease symptoms. Oospores are reported for the first time for *P. meconopsidis* from Asian collections on *Papaver somniferum*. Morphological descriptions, illustrations and a key are provided for all described *Peronospora* species on *Papaver*. *cox1* and *cox2* sequence data are confirmed as equally good barcoding loci for reliable *Peronospora* species identification, whereas ITS rDNA does sometimes not resolve species boundaries. Molecular phylogenetic data reveal high host specificity of *Peronospora* on *Papaver*, which has the important phytopathological implication that wild *Papaver* spp. cannot play any role as primary inoculum source for downy mildew epidemics in cultivated opium poppy crops.

## Introduction

The genus *Papaver* (*Papaveraceae*) comprises about 80 annual, biennial and perennial herbs distributed in Central and south-western Asia, Central and Southern Europe and North Africa [1]. The most well-known species is opium or oilseed poppy (*P. somniferum*), an ancient crop and medicinal plant cultivated for its edible seed as well as for the production of opium, the source for important pharmaceutical drugs including morphine, thebaine, codeine, papaverine, and noscapine [2].

One of the most important diseases of *P. somniferum* is downy mildew caused by *Peronospora* spp., which is responsible for substantial crop losses world-wide (e.g. [2]–[6]). Several *Papaver* species have been reported to be hosts of *Peronospora* [7], and four species have been described from various *Papaver* species [8]. However, their taxonomic status, synonymy as well as host range have been much disputed, leading to substantial confusion in the literature about the number of species present on *Papaver* and their correct naming.

The most well-known species of *Peronospora* on *Papaver* and closely related host genera is *P. arborescens*, which was originally described from *Papaver rhoeas* by Berkeley [9]. Subsequently it has been also reported from numerous other hosts like *Argemone mexicana* [10], several *Meconopsis* spp. including *M. betonicifolia*, *M. cambrica*, *M. latifolia*, *M. napaulensis*, *M. polyanthemos* and *M. simplicifolia* [7], [11]–[14], and *Papaver* spp. including *P. alpinum*, *P. argemone*, *P. caucasicum*, *P. dubium*, *P. hybridum*, *P. lecoqii*, *P. litwinowii*, *P. nudicaule*, *P. orientale*, *P. pavoninum*, *P. setigerum* and *P. somniferum* [3], [4], [7], [11]–[13], [15]–[23].

*Peronospora cristata*, the second species described from *Papaver*, was reported to infect *P. argemone*, *P. hybridum*, *P. rhoeas* and *P. somniferum* [5], [8], [13], [14], [17], but also *Meconopsis betonicifolia* [24] and *M. cambrica* [14]. Remarkably, *P. cristata* has only been reported on host species that are also recorded hosts of *P. arborescens*, raising the question about correct species identification and whether one or two species are involved. In the description of *P. cristata*, Tranzschel [25] reported verrucose oospores which are remarkably distinct from the smooth oospores of *P. arborescens*, but following Reid [14] who did not mention the oospore characteristics this important character has been largely ignored, and accessions from various *Papaver* and *Meconopsis* species were attributed to *P. cristata* primarily on conidial sizes that are distinctly larger than those of *P. arborescens*.

From *M. cambrica*, a third species, *P. meconopsidis*, has been described [26], which, however, has not received much attention in the plant pathology literature and has been commonly synonymised with *P. cristata* due to conidia of similar size, or ignored, following Reid [14] who did not even mention *P. meconopsidis*. The fourth species, *P. argemones*, was described from *Papaver argemone* and has conidial sizes in the range of *P. cristata* and *P. meconopsidis*. Therefore, Reid [14] synonymised it with *P. cristata*, ignoring the fact that the oospores of *P. argemones* were described as smooth, in contrast to the verrucose oospores of *P. cristata*. Following the approach of Reid [14], two *Peronospora* species, *P. arborescens* and *P. cristata*, have been accepted on *Papaver* until recently, which were primarily distinguished on their different conidial sizes, and more recently, on distinct ITS sequences [5], [20].

For risk assessment of infections of the economically important opium poppy (*Papaver somniferum*) crop, it is crucial to clarify the host ranges of the pathogens involved. Furthermore, since high numbers of wild *Papaver* spp. are coincident with the phenology of the cultivated opium poppy, if there is a host overlap, those *Papaver* spp. could act as alternative hosts and be potential sources of primary inoculum for the disease contributing to disseminating the pathogen within opium poppy crops. In the current study, we report the results of extensive molecular and morphological investigations on *Peronospora* accessions from various *Papaver* species and from *Meconopsis cambrica* to clarify nomenclature, species boundaries and host ranges of the species involved.

## Materials and Methods

### Morphological Analysis

Conidiophores and conidia were removed from the underneath of infected leaves, transferred to a drop of anhydrous lactic acid on a slide, carefully torn apart using forceps and needles, shortly heated using an alcohol burner and covered with a cover slip. For oogonia, host tissue was soaked in 2% KOH on a slide, carefully torn apart with forceps and needles and covered with a cover slip. Slides were examined and photographed using a Zeiss Axio Imager.A1 (Zeiss, Jena, Germany) microscope equipped with a Zeiss AxioCam ICc3 digital camera. Measurements are reported as maxima and minima in parentheses and the mean plus and minus the standard deviation of a number of measurements given in parentheses.

### Sample Sources

Information on the samples used for sequencing and phylogenetic analyses is given in Table 1. Details on the specimens used for morphological analysis are given in the description of the species. The herbarium acronyms are given according to Thiers [27].

**Table 1:** Sources and GenBank accession numbers of *Peronospora* and *Pseudoperonospora* material used for molecular phylogenetic analyses.

						GenBank accession no.		
Taxon	Host	Geographic origins	Collector	Accession	Voucher	ITS	*cox1*	*cox2*
*Peronospora alsinearum*	*Stellaria media*	Austria, Niederösterreich, Prellenkirchen	H. Voglmayr	HV2572	WU 32433		**KJ651277**	**KJ651340**
*P. apula*	*Papaver apulum*	Croatia, Istrija, Bale, Mandriol	H. Voglmayr	HV2388	WU 32408	**KJ651404**	**KJ651278**	**KJ651341**
*P. apula*	*Papaver apulum*	Croatia, Istrija, Rovinj, Valalta	H. Voglmayr	HV2909	WU 32409	**KJ651405**	**KJ651279**	**KJ651342**
*P. apula*	*Papaver apulum*	Croatia, Istrija, Rovinj, Kamp Veštar	H. Voglmayr	HV2911	WU 32410	**KJ651406**	**KJ651280**	**KJ651343**
*P. arborescens*	*Papaver rhoeas*	Austria, Burgenland, Kittsee	H. Voglmayr	HV17	WU 22880	**KJ651407**	**KJ651281**	**KJ651344**
*P. arborescens*	*Papaver rhoeas*	Austria, Niederösterreich, Bad Vöslau	H. Voglmayr	HV2604	WU 32411	**KJ651408**	**KJ651282**	**KJ651345**
*P. arborescens*	*Papaver rhoeas*	Austria, Wien, Leopoldstadt, Praterspitz	H. Voglmayr	HV2823	WU 32412	**KJ651409**	**KJ651283**	**KJ651346**
*P. arborescens*	*Papaver rhoeas*	Croatia, Istrija, Peroj	H. Voglmayr	HV2917	WU 32413	**KJ651410**	**KJ651284**	**KJ651347**
*P. arborescens*	*Papaver rhoeas*	France, Drôme, Hameau des Balmes	H. Voglmayr	HV-F24	WU 32415	**KJ651411**	**KJ651285**	**KJ651348**
*P. arborescens*	*Papaver rhoeas*	France, Herault, Montbazin	H. Voglmayr	HV-F57	WU 32414		**KJ651286**	**KJ651349**
*P. arborescens*	*Papaver rhoeas*	Germany, Baden-Württemberg, Tübingen	H. Voglmayr	HV815	WU 32416	**KJ651412**	**KJ651287**	**KJ651350**
*P. arborescens*	*Papaver rhoeas*	Hungary, Hajdú-Bihar, Hajdúszoboszló	H. Voglmayr	HV2462	WU 32417	**KJ651413**	**KJ651288**	**KJ651351**
*P. arborescens*	*Papaver rhoeas*	Italy, Lombardia, Barzano, Arzenate	H. Voglmayr	HV2942	WU 32418	**KJ651414**	**KJ651289**	**KJ651352**
*P. arborescens*	*Papaver rhoeas*	Romania, Constan?a, Hagieni	G. Negrean		MA-Fungi 9164	EU570201*	**KJ651290**	**KJ651353**
*P. arborescens*	*Papaver rhoeas*	Spain, Toledo, Hormigos, Malpica de Tajo	B. B. Landa & M. Montes	R1		EU570203*	**KJ651291**	**KJ651354**
*P. arborescens*	*Papaver rhoeas*	Spain, Puerto de Canales	A. Gustavsson		MA-Fungi 27844	EU570197*	**KJ651292**	**KJ651355**
*P. arborescens*	*Papaver rhoeas*	Spain, Naroba, Quiviesa river	A. Gustavsson		MA-Fungi 27843	EU570199*	**KJ651293**	**KJ651356**
*P. argemones*	*Papaver argemone*	Germany, Sachsen-Anhalt, Friedersdorf	F. Jage	HV2427	GLM 64084	**KJ651415**	**KJ651294**	**KJ651357**
*P. arthurii*	*Oenothera biennis* agg.	Austria, Niederösterreich, Gmünd	H. Voglmayr	HV2298	WU 32434		**KJ651295**	**KJ651358**
*P. boni-henrici*	*Chenopodium bonus-henricus*	Austria, Tirol, Obertilliach	H. Voglmayr	HV639	WU 22886		**KJ651296**	**KJ651359**
*P. bulbocapni*	*Corydalis cava*	Austria, Wien, Landstraβe, Botanical Garden	H. Voglmayr	HV7	WU 22887		**KJ651297**	**KJ651360**
*P. chrysosplenii*	*Chrysosplenium alternifolium*	Austria, Oberösterreich, St. Willibald	H. Voglmayr	HV58	WU 22892		**KJ651298**	**KJ651361**
*P. conglomerata*	*Geranium molle*	Austria, Niederösterreich, Brunn an der Wild	H. Voglmayr	HV2678	WU 32435		**KJ651299**	**KJ651362**
*P. corydalis*	*Corydalis solida*	Austria, Niederösterreich, Mannersdorf/Leithageb.	H. Voglmayr	HV2157	WU 32436		**KJ651300**	**KJ651363**
*P. cristata*	*Papaver hybridum*	Spain, Málaga, Antequera, Lavadero	B. B. Landa & M. Montes	BL-C90			**KJ651301**	**KJ651364**
*P. cristata*	*Papaver hybridum*	Spain, Córdoba, IFAPA	B. B. Landa & M. Montes	BL-B25	WU 32419	**KJ651416**	**KJ651302**	**KJ651365**
*P. cristata*	*Papaver hybridum*	Spain, Córdoba, IFAPA	B. B. Landa & M. Montes	BL-C25	WU 32420	**KJ651417**	**KJ651303**	**KJ651366**
*P. cristata*	*Papaver hybridum*	Spain, Córdoba, IFAPA	B. B. Landa & M. Montes	BL-C75	WU 32421	**KJ651418**	**KJ651304**	**KJ651367**
*P. holostei*	*Holosteum umbellatum*	Hungary, Eger, Demjén	H. Voglmayr	HV2439	WU 32437		**KJ651305**	**KJ651368**
*P. lamii*	*Lamium purpureum*	Austria, Oberösterreich, Enzenkirchen	H. Voglmayr	HV2619	WU 32438		**KJ651306**	**KJ651369**
*P. meconopsidis*	*Meconopsis cambrica*	Austria, Steiermark, Graz	H. Voglmayr	HV2010	WU 32422	**KJ651419**	**KJ651307**	**KJ651370**
*P. meconopsidis*	*Meconopsis cambrica*	UK, London, Kew Gardens	H. Voglmayr	HV2360	WU 32423	**KJ651420**	**KJ651308**	**KJ651371**
*P. meconopsidis*	*Meconopsis cambrica*	UK, England	D. E. L. Cooke	P24MC		DQ885375*	**KJ651309**	**KJ651372**
*P. meconopsidis*	*Papaver pavoninum*	Turkmenistan, Kordon Kepelya	V. A. Melnik	HV2965	K(M) 179241	**KJ651421**	**KJ651310**	**KJ651373**
*P. meconopsidis*	*Papaver somniferum*	Australia, Tasmania	P.J. Cotterill	BL-Cot1			**KJ651311**	**KJ651374**
*P. meconopsidis*	*Papaver somniferum*	Australia, Tasmania	P.J. Cotterill	BL-Cot2			**KJ651312**	**KJ651375**
*P. meconopsidis*	*Papaver somniferum*	Australia, Tasmania	P.J. Cotterill	BL-Cot3			**KJ651313**	**KJ651376**
*P. meconopsidis*	*Papaver somniferum*	Australia, Tasmania	F. S. Hay	BL-Hay			**KJ651314**	**KJ651377**
*P. meconopsidis*	*Papaver somniferum*	Afghanistan, Jalalabad	M. A. Ghani	HV2963	K(M)179245	**KJ651422**	**KJ651315**	**KJ651378**
*P. meconopsidis*	*Papaver somniferum*	Austria, Niederösterreich, Zwerndorf	H. Voglmayr	HV2749	WU 32424	**KJ651423**	**KJ651316**	**KJ651379**
*P. meconopsidis*	*Papaver somniferum*	Austria, Oberösterreich, St. Willibald	H. Voglmayr	HV2190	WU 32425	**KJ651424**	**KJ651317**	**KJ651380**
*P. meconopsidis*	*Papaver somniferum*	Czech Republic, Morava, Teplice nad Bečvou	H. Voglmayr	HV2728	WU 32426	**KJ651425**	**KJ651318**	**KJ651381**
*P. meconopsidis*	*Papaver somniferum*	Pakistan, Darra Adam Khel	M. A. Ghani	HV2966	K(M) 179248	**KJ651426**	**KJ651319**	**KJ651382**
*P. ranunculi*	*Ranunculus repens*	Austria, Steiermark, Mitterberg	H. Voglmayr	HV2293	WU 32439		**KJ651320**	**KJ651383**
*P. somniferi*	*Papaver somniferum*	Austria, Oberösterreich, Kronsdorf	G. Bedlan	HV2972	WU 32427	**KJ651427**	**KJ651321**	**KJ651384**
*P. somniferi*	*Papaver somniferum*	Czech Republic, Morava, Teplice nad Bečvou	H. Voglmayr	HV2726	WU 32428	**KJ651428**	**KJ651322**	**KJ651385**
*P. somniferi*	*Papaver somniferum*	Finland, Lepola, Pornainen	J. I. Liro		MA-Fungi 28507	EU570200*	**KJ651323**	**KJ651386**
*P. somniferi*	*Papaver somniferum*	Spain, Toledo, Torrejón	B. B. Landa & M. Montes	P18TVMT		DQ885370*	**KJ651324**	**KJ651387**
*P. somniferi*	*Papaver somniferum*	Spain, Toledo, Torrejón	B. B. Landa & M. Montes	P21TTMT		DQ885373*	**KJ651325**	**KJ651388**
*P. somniferi*	*Papaver somniferum*	Spain, Sevilla, Marchena, Cortijo del Río	B. B. Landa & M. Montes	BL-B8	WU 32432	**KJ651429**	**KJ651326**	**KJ651389**
*P. somniferi*	*Papaver somniferum*	Spain, Málaga, Antequera, El Pontón	B. B. Landa & M. Montes	BL-B5			**KJ651327**	**KJ651390**
*P. somniferi*	*Papaver somniferum*	Spain, Antequera, Valsequillo	B. B. Landa & M. Montes	P27MAV		DQ885376*	**KJ651328**	**KJ651391**
*P. somniferi*	*Papaver somniferum*	Spain, Écija, Casilla S. José	B. B. Landa & M. Montes	P6ESJ	WU 32430	DQ885363*	**KJ651329**	**KJ651392**
*P. somniferi*	*Papaver somniferum*	Spain, Écija, Viso Alto	B. B. Landa & M. Montes	P7EVA		DQ885364*	**KJ651330**	**KJ651393**
*P. somniferi*	*Papaver somniferum*	Spain, Albacete, Casa de los Llanos	B. B. Landa & M. Montes	P13ACALL	WU 32429	DQ885367*	**KJ651331**	**KJ651394**
*P. somniferi*	*Papaver somniferum*	Spain, Toledo, Valdemerino	B. B. Landa & M. Montes	P16TVMT		DQ885368*	**KJ651332**	**KJ651395**
*P. somniferi*	*Papaver somniferum*	Spain, Burgos	B. B. Landa & M. Montes	BL-BuB3		**KJ651430**	**KJ651333**	**KJ651396**
*P. sordida*	*Scrophularia nodosa*	Austria, Kärnten, St. Margareten im Rosental	H. Voglmayr	HV2395	WU 32440		**KJ651334**	**KJ651397**
*P.* sp. 1	*Papaver dubium*	Poland, Nowy Dwór Gdański, Kąty Rybackie	J. Kochman	HV2961	K(M) 94856	**KJ651431**		**KJ651398**
*P.* sp. 1	*Papaver dubium*	Spain, Puerto de San Glorio	A. Gustavsson		MA-Fungi 27841	EU570198*	**KJ651335**	**KJ651399**
*P.* sp. 2	*Papaver* sp.	Romania, Oltenia, Craiova	M. Costescu	HV2962	K(M) 179240	**KJ651432**	**KJ651336**	**KJ651400**
*P. trivialis*	*Cerastium holosteoides*	Austria, Oberösterreich, Kopfing	H. Voglmayr	HV2618	WU 32441		**KJ651337**	**KJ651401**
*Pseudoperonospora cannabina*	*Cannabis sativa*	Austria, Niederösterreich, Rauchenwarth	H. Voglmayr	HV2740	WU 32442		**KJ651338**	**KJ651402**
*Pseudoperonospora cubensis*	*Echinocystis lobata*	Austria, Niederösterreich, Markthof	H. Voglmayr	HV2776	WU 32443		**KJ651339**	**KJ651403**

For institution codes of herbarium vouchers see Thiers [27]; asterisks (*) denote ITS sequences published in Landa et al. [4] and Montes-Borrego et al. [39]; all others were newly generated in the present study (formatted in bold).

### DNA Extraction, PCR and Sequencing

For DNA extraction, infected dry host tissue was placed in 2 ml reaction tubes together with six sterile 2 mm glass beads and ground in a Retsch 200 mixer mill for 10 min. Alternatively, conidiophores were scraped off the leaf surface of the hosts, put in 1.5 µl reaction tubes and ground with sterile quartz sand and a conical micropestle. DNA was extracted using the modified CTAB protocol described in Riethmüller et al. [28] or using the Macherey-Nagel NucleoSpin Plant II extraction kit according to the manufacturer's instructions.

A ca. 2200 long fragment containing partial nuSSU-ITS-LSU rDNA was amplified using primers DC6 [29] and LR6-O [28] or LR6-O1 (designed here; 5' CGCATCGCCAGACGAGC 3'). In cases where no product could be obtained, the ITS was amplified using primers DC6 and ITS4 [30]. For cycle sequencing, primers ITS5-P (designed here; 5' GGAAGGTGAAGTCGTAACAAGG 3'), ITS4, LR0R [31] and LR6-O were used. For the mitochondrial cytochrome c oxidase subunit I (*cox1*) sequences, primers Oom-CoxI-lev-up and Oom-CoxI-lev-lo [32] were used for amplification and cycle sequencing; the cytochrome c oxidase subunit II (*cox2*) was amplified and cycle-sequenced with the forward and reverse primers of Hudspeth et al. [33]. The PCR products were purified using an enzymatic PCR cleanup [34] according to the protocol of Voglmayr and Jaklitsch [35]. DNA was cycle-sequenced using the ABI PRISM Big Dye Terminator Cycle Sequencing Ready Reaction Kit v. 3.1 (Applied Biosystems, Warrington) and an automated DNA sequencer (AB 3730xl Genetic Analyzer, Applied Biosystems).

### Phylogenetic Analysis

All alignments were produced with Muscle version 3.6 [36]. For evaluation of species status, a combined analysis of *cox1* and *cox2* was performed, adding a representative selection of *Peronospora* species according to the phylogenetic tree of Göker et al. [37], with two *Pseudoperonospora* species as outgroup to root the tree according to the phylogenies of Göker et al. [38]. Because the ITS-LSU rDNA did not show much phylogenetic information to separate closely related species, it was not included in the phylogenetic analyses but the sequences were deposited in GenBank (Table 1); in addition, for complete reference the GenBank accession numbers of ITS sequences of *Peronospora* accessions included in the present study that have already been deposited by the authors in the course of previous studies [4], [39] are also listed in Table 1.

Maximum parsimony (MP) analysis was performed with PAUP* version 4.0 b10 [40], using 1000 replicates of heuristic search with random addition of sequences and subsequent TBR branch swapping (MULTREES option in effect, COLLAPSE = MAXBRLEN, steepest descent option not in effect). All molecular characters were unordered and given equal weight; analyses were performed with gaps treated as missing data. Bootstrap analysis with 1000 replicates was performed in the same way, but using 5 rounds of random sequence addition and subsequent branch swapping during each bootstrap replicate.

For maximum likelihood (ML) and Bayesian analyses, the HKY substitution model [41] was selected for both *cox1* and *cox2* by Modeltest 3.6 [42] using the hierarchical likelihood ratio tests, with invariant sites and gamma distribution for the remaining sites (HKY+I+G). In the combined analyses, substitution parameters were estimated separately for each region. For ML analyses, 10 runs with 100 thorough bootstrap replicates each were computed with RAxML [43] as implemented in raxmlGUI 1.3 [44] using the GTRCAT substitution model. For Bayesian analyses using MrBayes version 3.1.2 [45], three parallel runs of four incrementally heated simultaneous Markov chains were performed over 1 million generations from which every 100th tree was sampled in each run, implementing the HKY+I+G substitution model.

### Nomenclature

The electronic version of this article in Portable Document Format (PDF) in a work with an ISSN or ISBN will represent a published work according to the International Code of Nomenclature for algae, fungi, and plants, and hence the new names contained in the electronic publication of a PLOS ONE article are effectively published under that Code from the electronic edition alone, so there is no longer any need to provide printed copies.

In addition, new names contained in this work have been submitted to MycoBank from where they will be made available to the Global Names Index. The unique MycoBank number can be resolved and the associated information viewed through any standard web browser by appending the MycoBank number contained in this publication to the prefix http://www.mycobank.org/MB/. The online version of this work is archived and available from the following digital repositories: PubMed Central, LOCKSS.

## Results

The final matrix was deposited in TreeBASE (http://www.treebase.org) and is available under http://purl.org/phylo/treebase/phylows/study/TB2:S15609.

**Figure 1:**
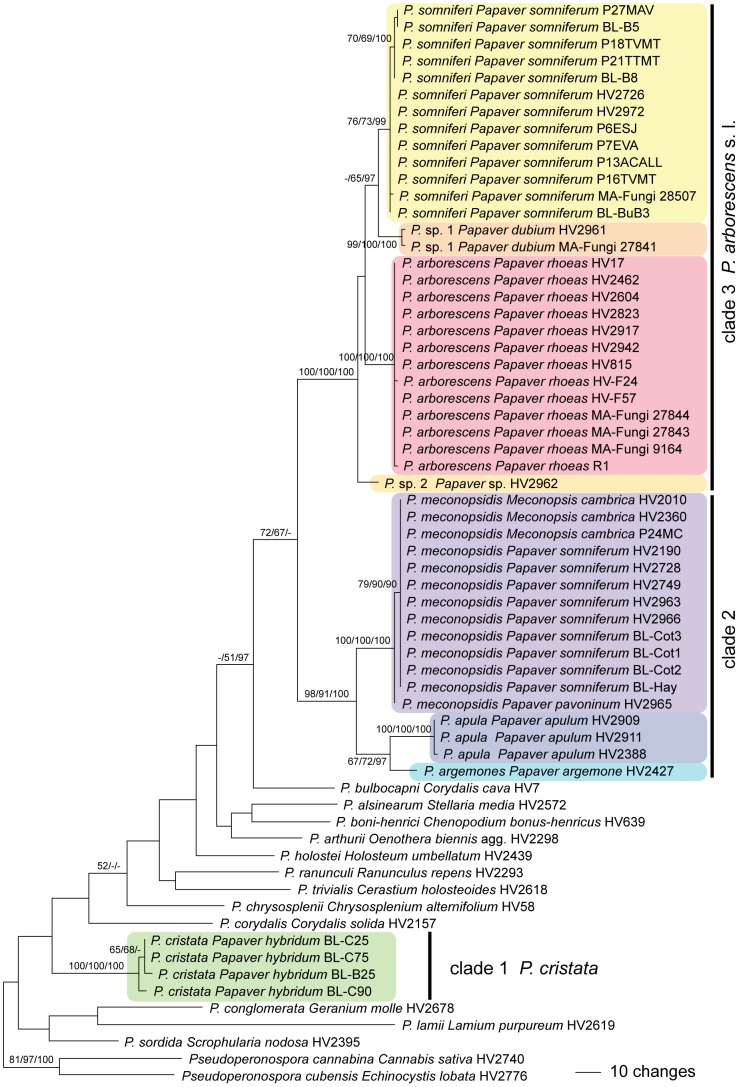
Phylogram showing phylogenetic relationships of *Peronospora* accessions from *Papaver* and *Meconopsis*. One of 16 most parsimonious trees 1035 steps long inferred from the combined *cox1*-*cox2* sequence data matrix; parsimony and likelihood bootstrap support above 50% and posterior probabilities above 90% are given at first, second and third position, respectively, above/below the branches. The tree was rooted with two species of *Pseudoperonospora* according to Göker et al. [38].

Of the 1262 characters of the combined *cox1* - *cox2* alignment, 234 were parsimony informative (120 in *cox1*, 114 in *cox2*). MP analyses revealed 16 MP trees 1035 steps long, one of which was selected and presented in Figure 1, with MP and ML bootstrap support above 50% and posterior probabilities above 90% given at first, second and third position, respectively, above/below the branches. Topologies of the MP trees slightly differed in the phylogenetic positions of *P. sordida*, *P. corydalis* and *P. chrysosplenii*. The three Bayesian runs revealed almost identical posterior probabilities (PP) and were fully compatible with the MP strict consensus tree. The *Peronospora*-accessions from *Papaver*/*Meconopsis* were contained within three highly supported clades, one comprising *Peronospora cristata* from *Papaver hybridum* (clade 1 in Figure 1); a second clade containing *P. apula* from *Papaver apulum*, *P. argemones* from *Papaver argemones* and *P. meconopsidis* from *Meconopsis cambrica*, *Papaver pavoninum* and *P. somniferum* (clade 2 in Figure 1); and a third clade with *P. arborescens* from *Papaver rhoeas*, *P. somniferi* from *Papaver somniferum*, *P.* sp. 1 from *Papaver dubium* and *P.* sp. 2 from *Papaver* sp. (clade 3 in Figure 1). Whereas within the *Peronospora arborescens* clade the ITS did not resolve accessions from the different hosts, in the *cox1* - *cox2* trees the accessions from *Papaver dubium*, *P. rhoeas* and *P. somniferum* were placed in three distinct monophyletic clades, the former two with high and the latter with medium to high support (Figure 1).

Within the *Peronospora arborescens* sensu lato clade (clade 3 in Figure 1), *Peronospora* accessions from *Papaver somniferum* consistently exhibited 11 and 10 common sequence substitutions in *cox1* and *cox2*, respectively, if compared to sequences of *P. arborescens* from *Papaver rhoeas*, rendering them molecularly clearly distinct. *Peronospora* accessions from *Papaver somniferum* also differed morphologically from those from *P. rhoeas* in larger conidia (mean 21.1 × 17.7 µm vs. 18.3 × 16.1 µm).

### Taxonomy

As a result of the morphological and molecular phylogenetic investigations, six taxa are here recognised as occurring on *Papaver*, two of which are described as new. In addition, detailed descriptions of the other four already described species are provided. All type specimens cited were morphologically investigated in the present study.

### Peronospora Apula Voglmayr, sp. nov. Figure 2

Mycobank: MB 808433.

#### Description

*Infection* systemic or localized, when systemic whole plants or leaves stunted, stems strongly distorted, sinuous. *Down* mostly hypophyllous, greyish, consisting of dense and felt-like conidiophores. *Conidiophores* hyaline, straight to slightly sinuous, (170–)270–430(–500) µm long; trunk straight or curved, (60–)150–280(–360) µm long (n = 25), variable in width, 4–10.5 µm wide; callose plugs absent; upper part monopodially or subdichotomously branched 5–6 times. *Branches* curved, sinuous. *Ultimate branchlets* in pairs, straight to slightly curved, (3–)5.5–11.5(–19.5) µm long, 2–2.5 µm wide at the base (n = 349), apex obtuse. *Conidia* subhyaline to pale brown, subglobose, ellipsoidal to obovate, (14–)16.5–20(–22) µm long, (12–)14.5–17(–19) µm wide, mean 18.3 × 15.8 µm, l/w ratio (1.01–)1.07–1.25(–1.43) (n = 214), greatest width median, base and tip round; pedicel absent in most conidia but a scar visible at the point of attachment; producing germ tubes. *Oogonia* globose, subglobose to irregular, yellow brown to dark reddish brown, (29–)40–48(–54) µm diam., wall smooth, 1.5–2.7 µm thick (n = 88). *Oospores* distinctly aplerotic, globose, (21–)25–30(–34) µm diam., wall 1.9–4.5 µm thick (n = 88), smooth.

**Figure 2:**
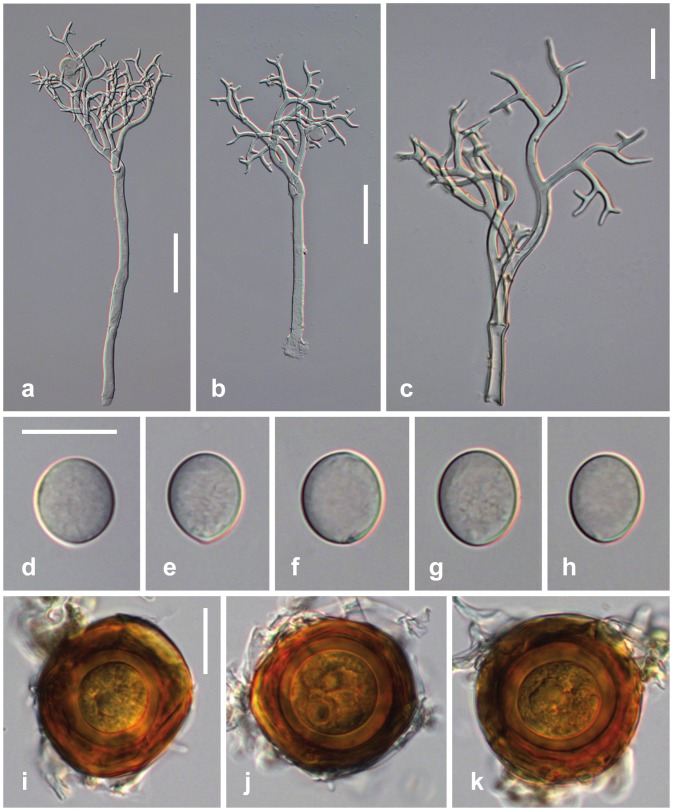
Morphological features of *Peronospora apula*. a, b conidiophores; c ultimate branchlets; d–h conidia; i–k oogonia and oospores. Sources: a, e–k WU 32410, holotype; b WU 32408; c, d WU 32409. Scale bars a, b 50 µm, c–k 20 µm.

#### Molecular diagnosis

*Peronospora apula* differs from its closest phylogenetic neighbour, *P. argemones*, by unique fixed alleles in two tree loci (*cox1*, *cox2*) based on alignments of the separate loci deposited in TreeBASE as study S15609: *cox1* positions 343: A; 49, 205, 346, 349, 425, 640: C; 148, 610, 664: G; 193, 217, 340, 652: T; *cox2* positions 154, 232, 370, 565: A; 295, 469: C; 65, 109, 187, 372, 373, 449: G; 105, 133, 262: T.

#### Etymology

Referring to its host, *Papaver apulum* Ten.

#### Habitat

On living leaves and stems of *Papaver apulum*.

#### Holotype

CROATIA, Istrija, SE Rovinj, ca. 800 m ESE Kamp Veštar, field, 18 May 2012, H. Voglmayr HV2911(WU 32410).

#### Additional specimens examined

CROATIA, Istrija, N Rovinj, E Valalta, 17 May 2012, H. Voglmayr HV2911 (WU 32409). Istrija, Mandriol ESE Bale, 14 May 2010, H. Voglmayr HV2388 (WU 32408).

#### Comments

*Peronospora apula* appears to be confined to *Papaver apulum*. It is closely related to *P. argemones* which differs by larger conidia, its host *Papaver argemone* and by different ITS (10 substitutions), *cox1* (14 substitutions) and *cox2* (15 substitutions) sequences.

**Figure 3:**
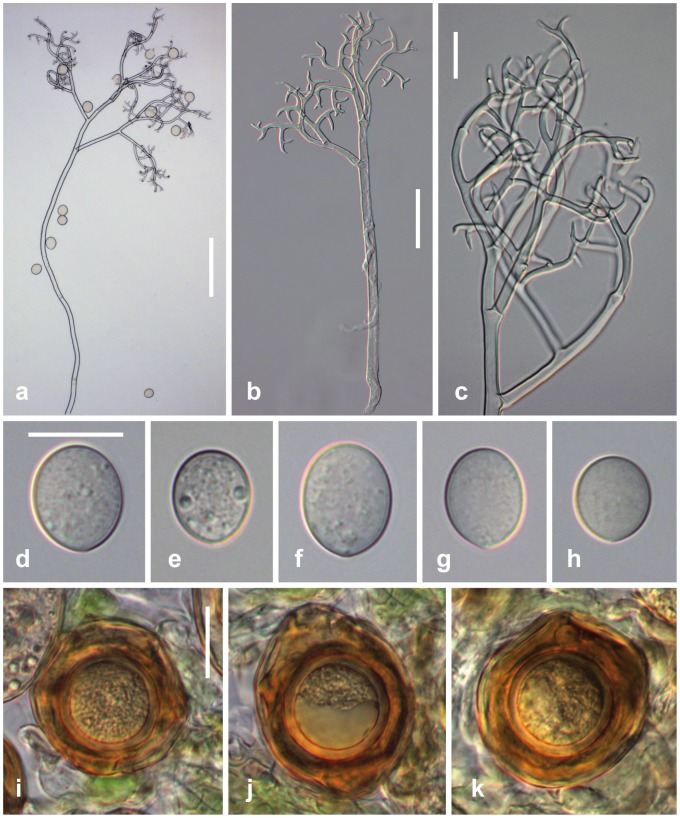
Morphological features of *Peronospora arborescens*. a, b conidiophores; c ultimate branchlets; d–h conidia; i–k oogonia and oospores. Sources: a WU 32416; b, k WU 32411; c, f, g WU 32412; d, h WU 32418; e WU 32413. Scale bars a 100 µm, b 50 µm, c–k 20 µm.

***Peronospora arborescens*** **(Berk.) de Bary, Monatsber. Königl. Preuss. Akad. Wiss. Berlin: 308-333 (1855)** **Figure 3** **.**

Basionym: *Botrytis arborescens* Berk., J. hort. Soc., London 1∶31 (1846).

#### Description

*Infection* commonly systemic, more rarely localized, when systemic whole plants or leaves stunted, strongly distorted, chlorotic, dwarfed, when localized producing polyangular to confluent lesions with distinct margins. *Down* hypophyllous, greyish, consisting of dense and felt-like conidiophores. *Conidiophores* hyaline, straight to slightly sinuous, (290–)360–600(–720) µm long; trunk straight or curved, (110–)190–380(–460) µm long (n = 23), variable in width, 5.5–13 µm wide; callose plugs absent; upper part monopodially or subdichotomously branched 5–7 times. *Branches* distinctly curved, sinuous. *Ultimate branchlets* mostly in pairs, slightly to strongly curved, (2–)4.5–9.5(–18) µm long, 2–2.7 µm wide at the base (n = 511), apex obtuse. *Conidia* subhyaline to pale brown, subglobose, ellipsoidal to obovate, (14–)16.5–20(–24) µm long, (12.5–)15–17.5(–20) µm wide, mean 18.3 × 16.1 µm, l/w ratio (1.01–)1.07–1.21(–1.45) (n = 413), greatest width median, base and tip round; pedicel absent in most conidia but a scar visible at the point of attachment; producing germ tubes. *Oogonia* globose, subglobose to irregular, yellow brown to dark reddish brown, (37–)43–51(–59) µm diam., wall commonly wrinkled, smooth, ca. 1 µm thick (n = 193). *Oospores* distinctly aplerotic, globose, (21–)26–30(–33) µm diam., wall 1.5–2.7 µm thick (n = 193), smooth.

#### Habitat

On living leaves and stems of *Papaver rhoeas* L.

#### Typification

UK, King's Cliffe, without date and collector, ex Herb. Berkeley (K(M) 178950, holotype).

#### Selected additional specimens examined

AUSTRIA, Burgenland, Neusiedl/See, Kittsee, E Groβer Raubwald near Edelstal, 27 Mar 1999, H. Voglmayr HV17 (WU 22880). Niederösterreich, Baden, Bad Vöslau, 19 Apr 2011, H. Voglmayr HV2604 (WU 32411). Wien, Leopoldstadt, Praterspitz, 4 Apr 2012, H. Voglmayr HV2823 (WU 32412). CROATIA, Istrija, E Peroj, 20 May 2012, H. Voglmayr HV2917 (WU 32413). FRANCE, Herault, SW Montpellier, between Montbazin and Cournosec, 9 Apr 2001, H. Voglmayr HV-F57 (WU 32414). S Lyon, Drôme, Hameau des Balmes W Romans-s-Isère, 7 Apr 2001, H. Voglmayr HV-F24 (WU 32415). GERMANY, Baden-Württemberg, Tübingen, E Lustnau, Furtwiesen, 28 Apr 2001, H. Voglmayr HV815 (WU 32416). HUNGARY, Hajdú-Bihar, between Hajdúszoboszló and Kaba, 25 Oct 2010, H. Voglmayr HV2462 (WU 32417). ITALY, Lombardia, NW Bergamo, Barzano, near Arzenate, 28 May 2012, H. Voglmayr HV2942 (WU 32418).

#### Comments

*Peronospora arborescens* appears to be confined to *Papaver rhoeas*, on which it is very common. Its disease symptoms are highly distinctive, infected plants being distorted and showing light yellowish green discolouration. The various accessions included in our molecular phylogenetic analyses covered most of Europe (Austria, Croatia, France, Germany, Hungary, Italy, Romania, Spain), and were molecularly highly homogeneous. Accessions from other *Papaver* species were placed in distinct clades and represent genetically distinct lineages. The accessions from Spain were partly sampled within cultivated opium poppy fields.

**Figure 4:**
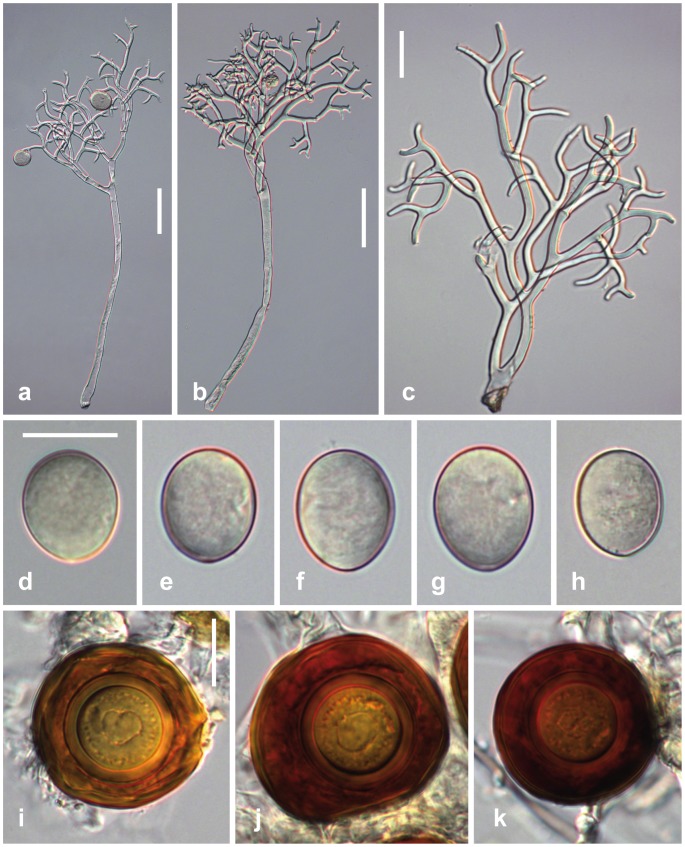
Morphological features of *Peronospora argemones*. a, b conidiophores; c ultimate branchlets; d–h conidia; i–k oogonia and oospores. Sources: a, c GLM 64084; b K(M) 181196, holotype. Scale bars a, b 50 µm, c-k 20 µm.

***Peronospora argemones*** **Gäum., Beitr. Kryptfl. Schweiz 5(no. 4): 72 (1923)** **Figure 4** **.**

#### Description

*Infection* commonly systemic, whole plants or leaves stunted, slightly to strongly distorted. *Down* hypophyllous, greyish, consisting of dense and felt-like conidiophores. *Conidiophores* hyaline, straight to slightly sinuous, (220–)290–490(–590) µm long; trunk straight to slightly curved, (80–)150–300(–390) µm long (n = 35), variable in width, 4–13 µm wide; callose plugs absent; upper part monopodially or subdichotomously branched 5–6 times. *Branches* tightly intertwined, curved, sinuous. *Ultimate branchlets* in pairs, slightly curved, (2.5–)6–14(–23) µm long, 1.8–2.8 µm wide at the base (n = 468), apex obtuse. *Conidia* subhyaline to pale brown, subglobose, ellipsoidal to obovate, (16.5–)19–23.5(–26) µm long, (14.5–)16.5–20(–23) µm wide, mean 21.1 × 18.1 µm, l/w ratio (1.02–)1.1–1.23(–1.37) (n = 181), greatest width median, base and tip round; pedicel absent in most conidia but a scar visible at the point of attachment; producing germ tubes. *Oogonia* globose, subglobose to irregular, light to dark reddish brown, (42–)48–59(–66) µm diam., wall smooth, 1–1.7 µm thick (n = 109). *Oospores* distinctly aplerotic, globose, (25–)28–34(–38) µm diam., wall 1.8–4.2 µm thick (n = 109), smooth.

#### Habitat

On living leaves and stems of *Papaver argemone* L.

#### Typification

GERMANY, Berlin, Lichterfelde, June 1896, P. Sydow, *Phycomyc. Protomyc. 5* (K(M) 181196, **lectotype here designated**, MBT177702; WU s.n., isotype).

#### Additional specimen examined

GERMANY, Sachsen-Anhalt, Bitterfeld, SW Friedersdorf, Muldeaue, 8 May 2004, H. Jage (GLM 64084).

#### Comments

*Peronospora argemones* appears to be confined to *Papaver argemone*. Gäumann [13] separated *P. argemones* from *P. arborescens* based on larger conidia. Our measurements fit well those reported by Gäumann [13] (21.1 × 18.1 vs. 21 × 18.6). *P. argemones* is not closely related to *P. arborescens* but to *P. meconopsidis* which has larger conidia. Its closest relative, however, is *P. apula*, which differs by slightly smaller conidia and its host *Papaver apulum*.

**Figure 5:**
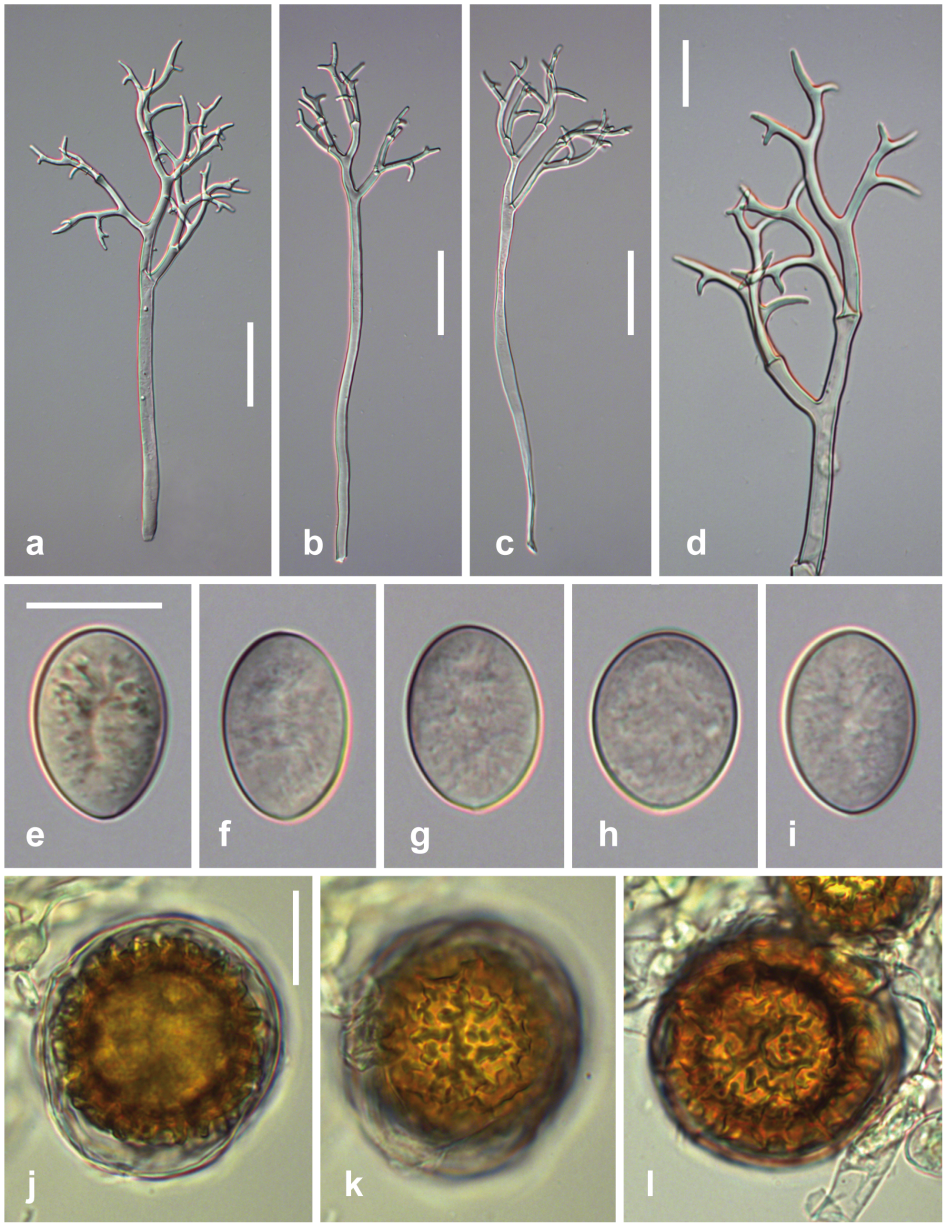
Morphological features of *Peronospora cristata*. a-c conidiophores; d ultimate branchlets; e–i conidia; j–l oogonia and oospores. Sources: a, b, d, e, j, k LE 185561, holotype; c, h WU 32419; f, g, i, l WU 32421. Scale bars a-c 50 µm, d-l 20 µm.

***Peronospora cristata*** **Tranzschel, Trav. Mus. Bot. Acad. Sci. St. Petersburg 1∶49 (1902)** **Figure 5** **.**

#### Description

*Infection* commonly systemic, more rarely localized, when systemic whole plants or leaves stunted, slightly distorted, chlorotic. *Down* hypophyllous, greyish, consisting of dense and felt-like conidiophores. *Conidiophores* hyaline, straight or slightly sinuously curved, (200–)240–330(–380) µm long; trunk straight or curved, (95–)130–220(–280) µm long (n = 33), variable in width, 4.5–10.5 µm wide; callose plugs absent; upper part monopodially or subdichotomously branched 3–5 times. *Branches* straight to slightly sinuously curved. *Ultimate branchlets* in pairs, slightly to distinctly curved, (2.5–)5–10.5(–19) µm long, 2–3.6 µm wide at the base (n = 263), apex obtuse. *Conidia* pale brown to brown, broadly ellipsoidal, ellipsoidal to obovate, (21.5–)25–28(–32) µm long, (15.5–)18–21(–24) µm wide, mean 26.5 × 19.6 µm, l/w ratio (1.15–)1.27–1.43(–1.53) (n = 122), greatest width median, base and tip round; pedicel absent in most conidia but a scar visible at the point of attachment; producing germ tubes. *Oogonia* mostly globose, rarely subglobose to irregular, golden to reddish brown, (38–)47–57(–69) µm diam., wall smooth, ca. 1 µm thick (n = 52). *Oospores* almost plerotic to slightly aplerotic, globose, (34–)40–49(–54) µm diam., wall distinctly irregularly verrucose, ca. 5.5–10 µm thick including ornamentation (n = 52); verrucae 1.2–2.3 µm thick, rarely isolated, commonly confluent, forming irregular crests to an incomplete labyrinthic net.

#### Habitat

On living leaves and stems of *Papaver hybridum* L.

#### Typification

UKRAINE, Crimea ("Tauria"), Georgyievskij Monastery near Sevastopol', 26 Apr 1901, W. Tranzschel (LE 185561, holotype).

#### Additional specimens examined

SPAIN, Córdoba, at (SW) the Puente de Andalucía, IFAPA, 4 Apr 2008, B. B. Landa & M. Montes (WU 32419). Same locality, 8 Apr 2008, B. B. Landa & M. Montes (WU 32420). Same locality, 15 Apr 2008, B. B. Landa & M. Montes (WU 32421). UKRAINE, Crimea ("Tauria"), Hortus Nikitensis, 26 Apr 1902, W. Tranzschel (LE 43379). Simeiz, 30 Apr 1902, W. Tranzschel (LE 43377). Jalta, 14 May 1903, W. Tranzschel (LE 185505).

#### Comments

*Peronospora cristata* has been confirmed only from *Papaver hybridum*, with which it is apparently co-occurring. The type and authentic specimens of Tranzschel [23] from the Crimean Peninsula morphologically fully resemble the recent Spanish collections for which DNA data are available. The irregularly verrucose oospores are highly distinctive for the species, all other *Peronospora* species on *Papaver* having smooth oospores. In addition, it has the largest conidia of all *Peronospora* species on *Papaver*. *Peronospora meconopsidis*, which has conidia of similar size, differs by smooth oospores and different hosts (*Papaver somniferum*, *Meconopsis cambrica*).

**Figure 6:**
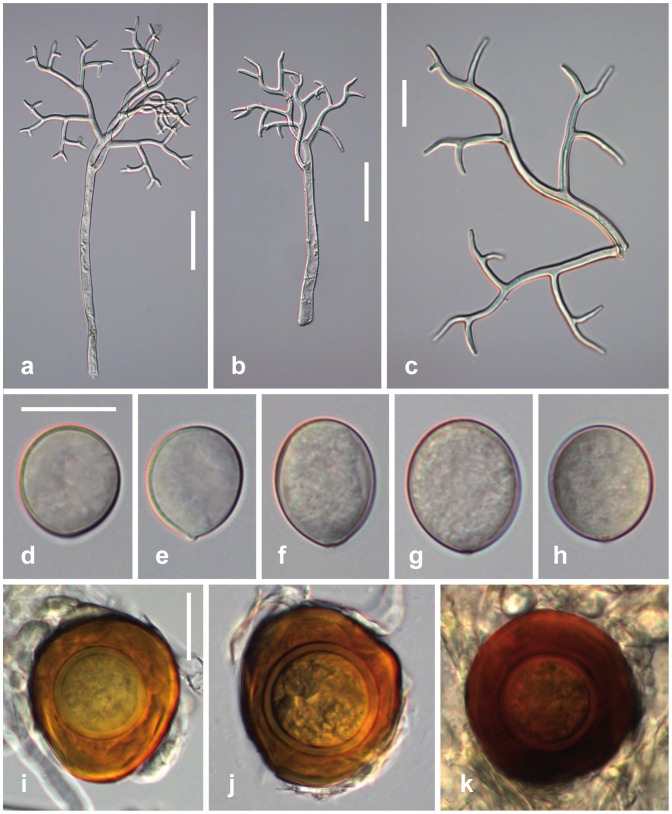
Morphological features of *Peronospora meconopsidis*. a, b conidiophores; c ultimate branchlets; d–h conidia; i–k oogonia and oospores. Sources: a, f, g WU 32426; b, c–e NEU, holotype; h WU 32425; i K(M) 179241; j, k K(M) 179245. Scale bars a, b 50 µm, c–k 20 µm.

***Peronospora meconopsidis*** **Mayor, Mém. Soc. neuchât. de Sc. Nat. 9(1): 34 (1958)** **Figure 6** **.**

#### Replaced synonym

*Peronospora gaeumannii* Mayor [as '*gaeumanni*'], Ber. schweiz. bot. Ges. 59∶278 (1949), non *Peronospora gaeumannii* Mundk. [as '*gaeumanni*'], Scientific Monogr. Coun. Agric. Res. India 12∶8 (1938).

#### Description

*Infection* localized, producing more or less distinct polyangular to confluent lesions. *Down* mostly hypophyllous, greyish, consisting of mostly scattered conidiophores, very rarely dense and felt-like. *Conidiophores* hyaline, straight, (230–)240–510(–730) µm long; trunk straight, (70–)110–330(–500) µm long (n = 39), variable in width, 5–15 µm wide; callose plugs absent; upper part monopodially or subdichotomously branched 3–5 times. *Branches* slightly to strongly sinuously curved. *Ultimate branchlets* in pairs, slightly to distinctly curved, (2.5–)7.5–17.5(–37) µm long, 1.9–3.3 µm wide at the base (n = 810), apex obtuse. *Conidia* pale to medium brown, subglobose, ellipsoidal to obovate, (17–)22.5–27.5(–35.5) µm long, (15–)19–22.5(–26) µm wide, mean 24.8 × 20.6 µm, l/w ratio (1.03–)1.13–1.28(–1.47) (n = 416), greatest width median, base and tip round; pedicel absent in most conidia but a scar visible at the point of attachment; producing germ tubes. *Oogonia* rarely globose, mostly subglobose to irregular, reddish brown, (29–)40–48(–54) µm diam., wall smooth, 1–1.5 µm thick (n = 88). *Oospores* distinctly aplerotic, globose, (21–)25–30(–34) µm diam., wall 1.7–3.3 µm thick (n = 88), smooth.

#### Habitat

On living leaves of Meconopsis cambrica Vig., Papaver pavoninum C.A. Mey. and P. somniferum L.

#### Typification

SWITZERLAND, Neuchâtel, Val-de-Travers, Môtiers, Gorges de la Poëta Raisse, on *Meconopsis cambrica*, 31 July 1945, 21 Sep 1946, 17 Sep 1947, 17 Aug 1949, 14 July 1952, E. Mayor (NEU, **lectotype here designated**, MBT177701). ibid., 17 Sep 1947, E. Mayor (NEU, isolectotype). Neuchâtel, Vallée des Ponts, Combe Varin, garden, 27 June 1920, E. Mayor (NEU, syntype).

#### Additional selected specimens examined

On *Meconopsis cambrica*: AUSTRIA, Styria, Graz, Botanical Garden, 14 Sep 2002, H. Voglmayr HV2010 (WU 32422). UK, London, Kew Gardens, 14 Nov 2008, H. Voglmayr HV2360 (WU 32423). On *Papaver pavoninum*: RUSSIA, Moskva, Principal Botanical Garden an SSSR, 1 June 1959, E. Protsenko (K(M) 179239). On *Papaver somniferum*: AFGHANISTAN, Jalalabad, 14 Mar 1973, M.A. Ghani 7 (K(M)179245). AUSTRIA, Niederösterreich, Gänserndorf, Weiden an der March, Dornparz ESE Zwerndorf, 6 July 2011, H. Voglmayr HV2749 (WU 32424). Austria, Oberösterreich, Schärding, St. Willibald, 31 July 2005, H. Voglmayr HV2190 (WU 32425). CZECH REPUBLIC, Morava, Hranice, between Teplice nad Bečvou and Černotin, 25 June 2011, H. Voglmayr HV2728 (WU 32426). PAKISTAN, Darra Adam Khel, 9 Mar 1975, M.A. Ghani (K(M) 179248). POLAND, Nisko, Rudnik, 2 July 1957, J. Kowalski, in *J. Kochman Mycoth. Polon. 11* (K(M) 179244). ROMANIA, Transsilvania, Hunedoara, Gura Zlata, 4 Aug 1963, M. Bechet, in *Borza, Gergely & Raţiu, Fl. Roman. Exs. 3006* (K(M) 179243). TURKMENISTAN, Kordon Kepelya, 18 Apr 1978, V.A. Mel'nik (K(M) 179241). THAILAND, Mae Cham, Chiang Mai ("Chiengmai"), 1 Sep 1982, P. Pitakpaivan 0472 (K(M) 179247).

#### Comments

*Peronospora meconopsidis* is commonly observed on *Meconopsis cambrica* and *Papaver somniferum*, but its disease symptoms are usually rather inconspicuous and localized compared to most other *Peronospora* species from *Papaver*. Although it has not been recorded from *Papaver somniferum* in Europe, it is apparently common and widespread on that host according to own observations and upon examination of herbarium specimens. It has commonly been misidentified as *Peronospora arborescens*, which mainly differs by conspicuous disease symptoms relating to mostly systemic infection which has never been observed for *P. meconopsidis*. Based on similar conidial sizes, *P. meconopsidis* has been referred to as *Peronospora cristata* in recent literature, which goes back to Reid [14] who classified accessions from *Meconopsis cambrica* under that species. However, *P. cristata* is easily distinguishable by its verrucose oospores, which are unique in *Peronospora* on *Papaver*. The Australia records of *Peronospora cristata* from *Papaver somniferum* [5] therefore actually represent *Peronospora meconopsidis*, which is also corroborated by sequence data (Figure 1).

Oospores of *Peronospora meconopsidis* are reported here for the first time, and they have only been found in young infected plants of *Papaver somniferum* collected in Asia. No oospores were found in specimens of *Papaver somniferum* from Europe or from *Meconopsis cambrica* despite thorough investigations.

The species was first described as *Peronospora gaeumannii* by Mayor [46]. However, because this is a younger homonym of *P. gaeumannii* Mundk., Mayor [26] proposed the new name *P. meconopsidis*. Three authentic specimens mentioned in the original description of *P. gaeumannii* Mayor are present at NEU, of which the largest, best developed and preserved is here selected as lectotype. This folder also contains the original drawings and spore statistics published in Mayor [46]. According to the herbarium label, the lectotype specimen consists of several collections from the same place collected from 1945 to 1952, which were subsequently mixed and cannot be separated any more. The mean spore sizes recorded by Mayor [46] agree well with those of the current study (23.5 × 21.15 vs. 24.8 × 20.6 µm).

**Figure 7:**
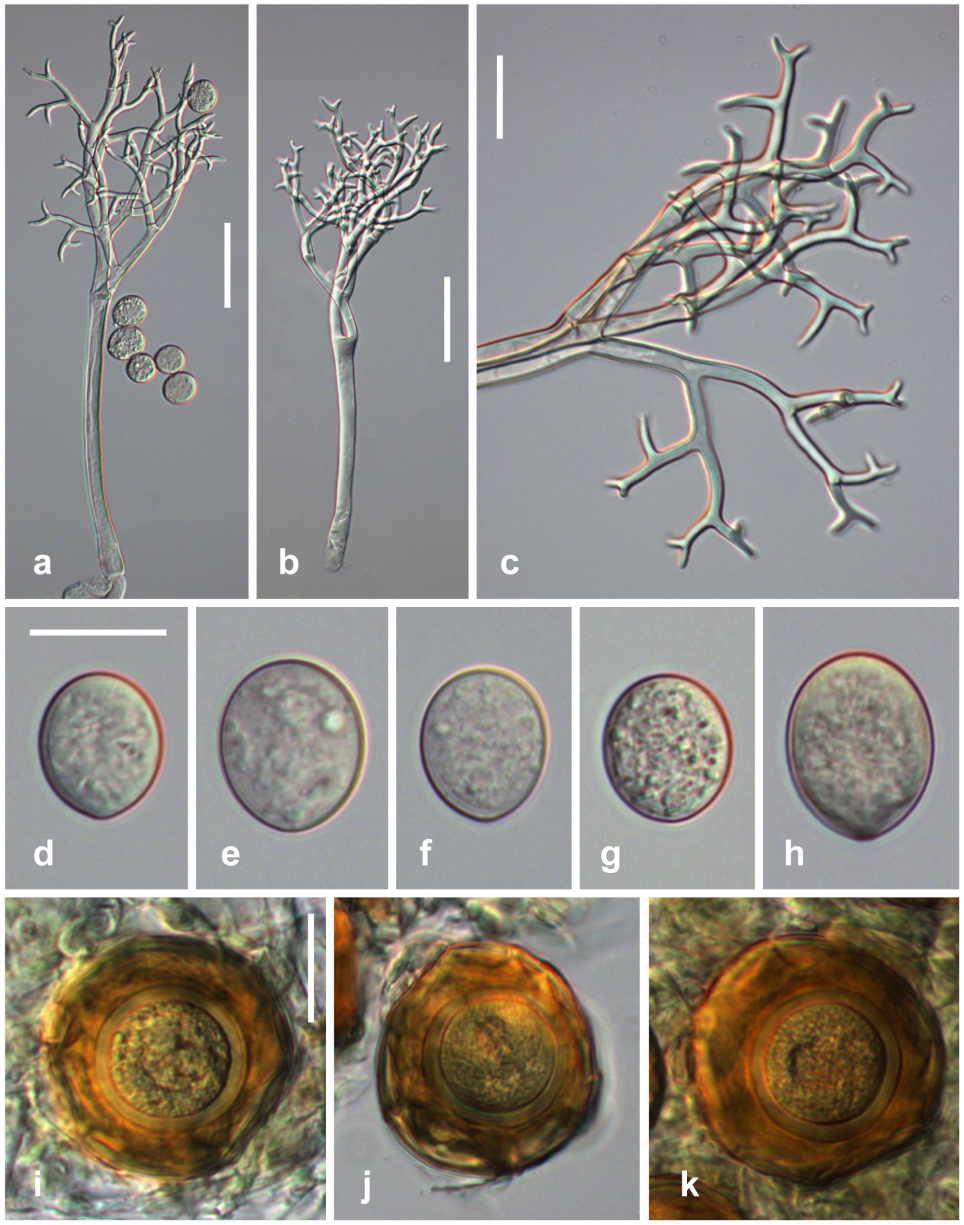
Morphological features of *Peronospora somniferi*. a, b conidiophores; c ultimate branchlets; d–h conidia; i–k oogonia and oospores. Sources: a, g WU 32432; b, d, i–k WU 32428, holotype; c MA 65574; e, f WU 32429; h WU 32430. Scale bars a, b 50 µm, c–k 20 µm.

***Peronospora somniferi*** **Voglmayr, sp. nov.** **Figure 7** **.**

Mycobank MB 808434.

#### Description

*Infection* systemic or localized, when systemic whole plants or leaves stunted, stems strongly distorted, sinuous. *Down* mostly hypophyllous, greyish, consisting of dense and felt-like conidiophores. *Conidiophores* hyaline, straight to slightly sinuous, (280–)320–510(–660) µm long; trunk straight or curved, (100–)140–330(–490) µm long (n = 33), variable in width, 5.5–17 µm wide; callose plugs absent; upper part monopodially or subdichotomously branched 4–7 times. *Branches* straight to sinuously curved. *Ultimate branchlets* in pairs, straight to slightly curved, (2–)4.5–10(–18.5) µm long, 1.9–3.2 µm wide at the base (n = 550), apex obtuse. *Conidia* subhyaline to pale brown, subglobose, ellipsoidal to obovate, (15.5–)19–23(–28) µm long, (14.5–)16.5–19(–22.5) µm wide, mean 21.1 × 17.7 µm, l/w ratio (1.01–)1.11–1.28(–1.48) (n = 927), greatest width median, base and tip round; pedicel absent in most conidia but a scar visible at the point of attachment; producing germ tubes. *Oogonia* globose, subglobose to irregular, yellow brown to dark reddish brown, (31–)40–48(–57) µm diam., wall smooth, ca. 1 µm thick (n = 157). *Oospores* distinctly aplerotic, globose, (19–)24–28(–34) µm diam., wall 1.6–2.8 µm thick (n = 157), smooth.

#### Molecular diagnosis

*Peronospora somniferi* differs from its closest phylogenetic neighbour, *P. arborescens*, by unique fixed alleles in two tree loci (*cox1*, *cox2*) based on alignments of the separate loci deposited in TreeBASE as study S15609: *cox1* positions 205, 337, 418, 491, 646: A; 195: C; 229, 331, 499, 557, 589: T; *cox2* positions 313, 370, 430, 523: A; 493: C; 382, 541: G; 220, 253, 427: T.

#### Etymology

Referring to its host, *Papaver somniferum* L.

#### Habitat

On living leaves and stems of *Papaver somniferum*.

#### Holotype

CZECH REPUBLIC, Morava, Hranice, between Teplice nad Bečvou and Černotin, 25 June 2011, H. Voglmayr HV2726 (WU 32428).

#### Additional specimens examined

AUSTRIA, Oberösterreich, Linz Land, Kronsdorf, Schieferegg, 25 May 2004, G. Bedlan 721 (WU 32427). IRAN, Neelabad, 10 Apr 1973, M.A. Ghani 3 (K(M) 179246). SPAIN, Albacete, Casa Arriba los Llanos, 10 June 2005, B. Landa (WU 32429). Sevilla, Écija, Casilla San José, 29 Apr 2004, B. Landa (WU 32430). Sevilla, Écija, San Rafael, 29 Apr 2004, B. Landa (WU 32431). Sevilla, Écija, Vacas, without collector and date (MA-Fungi 65574). Malaga, Antequera, Monteluna, without collector and date (MA-Fungi 65500). Marchena, Cortijo del Rio, 4 Apr 2004, B. Landa (WU 32432).

#### Comments

**Figure 8:**
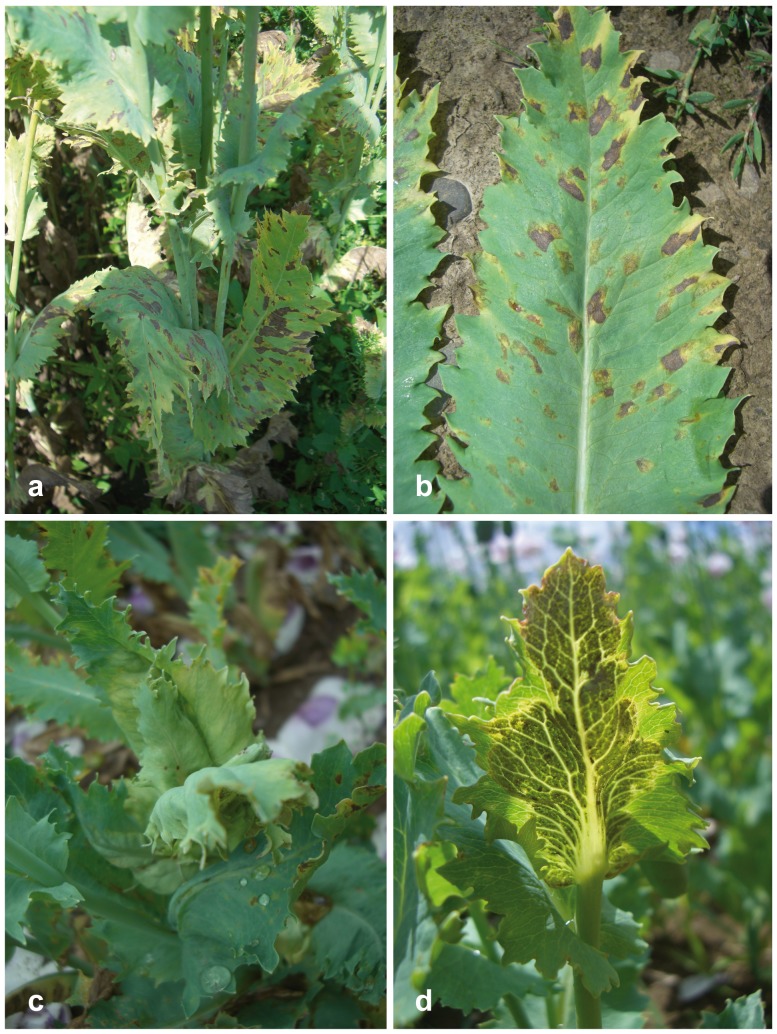
Disease symptoms of *Peronospora meconopsidis* and *P. somniferi* on opium poppy (*Papaver somniferum*). a, b polyangular leaf lesions of *P. meconopsidis*; c, d systemic infection of *P. somniferi* from above (c) and from underneath (d), showing the grey down. Sources: a WU 32424; b WU 32426; c, d WU 32428, holotype.

*Peronospora somniferi* appears to be confined to *Papaver somniferum*. It is closely related to *P. arborescens* which differs by smaller conidia and a different host, *Papaver rhoeas*. *Peronospora somniferi* cannot be reliably distinguished from *P. arborescens* by ITS data alone because there is only a single consistent nucleotide difference at the beginning of the ITS1, but *cox1* and *cox2* are distinctive and good barcode markers for the species (11 and 10 diagnostic substitutions, respectively). *Peronospora somniferi* has been reported as an economically important pathogen of *Papaver somniferum* throughout Europe [4]. *Peronospora meconopsidis*, which also occurs on *Papaver somniferum*, differs mainly by a non-systemic infection which is characterised by typical polyangular spots (see Figure 8).

### Key to known Species of *Peronospora* on *Papaver* and *Meconopsis*

Note: Morphological identification requires well developed material as well as knowledge of the host. For conidial measurements, it is crucial that a sufficient number of mature conidia are included. Because some species are highly similar, they often cannot be unequivocally identified by morphology alone, and sequence data (*cox1* or *cox2*) are essential in case of poorly developed specimens, if the host species is unknown or represents a host species from which no sequence data are available or from which several *Peronospora* species are known.

- 1 Oospore wall irregularly verrucose, only known from *Papaver hybridum...............................................................................* *P. cristata*
- Oospore wall smooth..................................................................2
- 2(1) Conidia in mean longer than 20 µm..................................3
- Conidia in mean shorter than 20 µm........................................5
- 3(2) Infection local, conidia in mean longer than 22 µm, confirmed from *Meconopsis cambrica*, *Papaver pavoninum*, *P. somniferum....................................................................* *P. meconopsidis*
- Infection systemic, conidia in mean shorter than 22 µm..........4
- 4(1) On Papaver argemone.....................................P. argemones
- On Papaver somniferum...........................................P. somniferi
- 5(2) On *Papaver apulum*, conidiophores (170–)270–430(–500) µm high...................................................................................*P. apula*
- On *Papaver rhoeas*, conidiophores (290–)360–600(–720) µm high............................................................................*P. arborescens*

## Discussion

### Molecular Phylogenetic Investigations

The current investigations clearly show that the biodiversity of *Peronospora* on *Papaver* is higher than previously perceived, which is in line with other investigations on *Peronosporaceae* (e.g. [37], [47]–[63]), demonstrating that high biodiversity is commonly the result of high host specificity. *Peronospora* from *Papaver* are distributed amongst three clades (Figure 1), of which *P. cristata* from clade 1 is phylogenetically isolated from the other *Peronospora* species from *Papaver*, but clades 2 and 3 appear to be closely related (Figure 1). Of special interest is clade 3, the species of which were formerly classified under *Peronospora arborescens*, and which is here referred to as *P. arborescens* sensu lato clade. Whereas the ITS data are highly similar within this clade and therefore do not allow for unequivocal distinction (data not shown), the *cox1* and *cox2* data are highly distinctive for the accessions from various hosts, in which each form genetically homogeneous lineages irrespective of the geographic origins. This is evident for the *Peronospora* accessions from *Papaver rhoeas* and *P. somniferum* which were sampled from various regions all over Europe, and which form distinct uniform genetic lineages. In addition, also differences in conidial sizes could be documented for both lineages. Therefore, the accessions from *Papaver somniferum*, previously classified under *P. arborescens*, are here described as a distinct species, *P. somniferi*. Apart from these two lineages, another two genetically distinct entities were present within the *P. arborescens* s. l. clade (Figure 1), *Peronospora* sp. 1 from *Papaver dubium*, and *Peronospora* sp. 2 from an unidentified *Papaver* species. However, because only few accessions were available for these, we currently refrain from describing them as new taxa. Considering the numerous additional *Papaver* species for which *Peronospora* have been recorded (see [4]) but for which no accessions were available for molecular phylogenetic investigations, additional genetically distinct entities may turn up in the future.

### Molecular Barcoding

*cox1*, chosen as barcoding locus for higher animals and considered to be the primary barcoding marker for organisms unless shown to be unsuitable (http://www.barcodeoflife.org), has been demonstrated to be an appropriate barcoding locus for oomycetes [32], which is confirmed in the current study. However, *cox2* shows similarly good resolution and consequently is an equally good barcoding marker; it even has some advantages over *cox1*, as it usually amplifies better especially in cases of low DNA quantity or old herbarium samples (as also shown in [64]), and thus *cox2* sequences are available for many more species in *Peronosporales*. The current study provides additional data for equally good discriminative power of *cox1* and *cox2*; e.g. *Peronospora somniferi* is distinct from *Peronospora arborescens* by shared 11 and 10 substitutions in *cox1* and *cox2*, respectively, clearly showing a similar significant barcode gap between both species in both markers. Therefore, both *cox1* and *cox2* are considered good markers for reliable identification of *Peronospora* species on *Papaver*, and it is recommended to sequence both loci in studies of *Peronosporales* whenever possible to obtain representative robust data for molecular barcoding. On the other hand, the ITS region does not resolve closely related species like *P. arborescens* and *P. somniferi*, which has been observed also in other groups of Peronosporaceae (e.g. [47], [54]).

### Nomenclature of *Peronospora* on *Papaver*

In the recent literature, accessions infecting *Papaver* were mainly classified as *Peronospora arborescens*. However, as the molecular data clearly show, there are several species involved, six of which are recognized and treated in the current publication. However, nomenclature of these species is partly complex mainly due to misleading species concepts of the past, as shall be outlined below.

In a phytopathological perspective, an important result of recent molecular phylogenetic investigations was that downy mildew disease of the crop *Papaver somniferum* is caused by two distinct species, which were classified as *Papaver arborescens* and *Papaver cristata* [4], [5]. However, the current detailed investigations show that this classification cannot be retained and has to be substantially modified.

Starting from Gäumann [13], *Peronospora* accessions from *Papaver somniferum* have been classified as *Peronospora arborescens*, which has been consistently followed. However, the current molecular phylogenetic investigations show that these accessions are genetically distinct from accessions from the type host, *Papaver rhoeas*, and there are also differences in conidial sizes. Consequently, the *Peronospora* accessions from *Papaver somniferum* belonging to the *Peronospora arborescens* sensu lato clade (clade 3; Figure 1) are here classified as a distinct species, *P. somniferi*.

Recently, the name *Peronospora cristata* was used for a second *Peronospora* species on *Papaver somniferum* that was first recorded from that host by Scott et al. [5] from Tasmania. This name was applied because their ITS sequences matched an English accession from *Meconopsis cambrica* that had previously been deposited in GenBank (DQ885375) under *Peronospora cristata*. This naming apparently goes back to Reid [14], who compared conidial measurements of *Peronospora* from *Meconopsis cambrica* with those from *Papaver argemone* and considered them to be conspecific due to similar size. In addition, because conidial sizes were also similar to those reported for *Peronospora cristata* from *Papaver hybridum*, he considered accessions from these three hosts to be conspecific, and thus classified them under *P. cristata* due to priority. However, he ignored that the oospores of *Peronospora cristata* are distinctly verrucose, being significantly different from the smooth oospores of *Peronospora argemones*. These substantial morphological differences are also mirrored by a distant phylogenetic position of *P. cristata* (Figure 1), confirming that *P. cristata* is a clearly distinct species confined to *Papaver hybridum*.

In addition, also the accessions from *Papaver argemone* and *Meconopsis cambrica* are phylogenetically distinct (Figure 1) and therefore have to be classified under *P. argemones* and *P. meconopsidis*, respectively. Consequently, as the *Peronospora* of *M. cambrica* and the second *Peronospora* species from *P. somniferum* are conspecific, the correct name to be applied for this species is *P. meconopsidis*.

### Host-parasite Relationships

The phylogenetic relationships of *Peronospora* on *Papaver* and those of their hosts are only partly congruent. For instance, within *Papaver* sect. *Argemonidium* which form a closely related group [1], the *Peronospora* species from *Papaver argemone* and *P. apulum* (*Peronospora argemones* and *P. apula*) are closest relatives, whereas the *Peronospora* from the third species of the section, *Papaver pavoninum*, falls within *P. meconopsidis* which is sister group to the former two species (Figure 1). However, *Peronospora cristata*, parasitising the fourth species of sect. *Argemonidium*, *Papaver hybridum*, is phylogenetically and morphologically distinct from all other *Peronospora* species on *Papaver*.

Remarkably, *Peronospora meconopsidis* infects hosts from two genera, *Meconopsis* and *Papaver*. However, molecular phylogenetic investigations show that *Meconopsis* is polyphyletic and embedded within *Papaver*, *M. cambrica* being unrelated to the other *Meconopsis* species [1]. Whereas the two main hosts of *Peronospora meconopsidis*, *M. cambrica* and *Papaver somniferum* are contained within the same clade but not closely related, the third host species, *P. pavoninum*, is member of the phylogenetically isolated *Papaver* section *Argemonidium* [1]. This indicates that phylogenetic radiation of *Peronospora* on *Papaver* is rather effected by host jumps.

Finally, from a pytopathological point of view the results from this study showed that wild *Papaver* spp. cannot play any role as primary inoculum for downy mildew epidemics in cultivated opium poppy crops since there is high host specificity. This result was corroborated when different *Peronospora* specimens (*P. somniferi*, *P. cristata* and *P. arborescens*) were sampled from the same fields of cultivated *Papaver somniferum* where *Papaver hybridum* and *Papaver rhoeas* were growing as weeds. Consequently, to avoid development of downy mildew epidemics in cultivated opium poppy efforts should be placed in controlling airborne *P. somniferi* sporangia from diseased opium poppy plants as well as avoiding the use infected seed lots [22].
